# Trends and disparities in skin malignant neoplasm mortality in the United States: a 22-year trend analysis based on CDC WONDER Data (1999-2020)

**DOI:** 10.3389/fonc.2026.1770375

**Published:** 2026-03-11

**Authors:** Yu Cao, Yun Li, Yuegang Wei

**Affiliations:** 1 NanJing University of Chinese Medicine, Nanjing, Jiangsu, China; 2 Department of Dermatology, The First Affiliated Hospital of Anhui University of Chinese Medicine, Hefei, Anhui, China

**Keywords:** CDC WONDER, epidemiology, joinpoint regression, mortality, skin malignant neoplasm, trend analysis

## Abstract

**Background:**

Skin malignant neoplasms are common malignancies worldwide, with epidemiological characteristics showing significant geographical and demographic disparities. This study aims to comprehensively analyze the temporal trends and demographic disparities in skin malignant neoplasm mortality among US adults from 1999 to 2020 using the CDC WONDER database.

**Methods:**

Mortality data for skin malignant neoplasms (ICD-10: C43-C44) among US adults (≥25 years) from 1999 to 2020 were obtained from the CDC’s WONDER online database. Joinpoint regression analysis was used to evaluate temporal trends in age-adjusted mortality rates (AAMR), calculating annual percent change (APC) and average annual percent change (AAPC). Stratified analyses were conducted by sex, age group, race/ethnicity, geographic region, and rural-urban status.

**Results:**

Between 1999 and 2020, a total of 251,848 skin malignant neoplasm deaths were reported in the United States, with an age-adjusted mortality rate of 5.299 per 100,000 person-years. Male mortality rates (8.137 per 100,000) were significantly higher than female rates (3.157 per 100,000). The overall mortality rate showed an increasing trend from 1999 to 2014 (APC =+ 0.271%), followed by a significant decrease from 2014 to 2017 (APC=-5.292%), and remained stable from 2017 to 2020 (APC=-0.563%).Age group analysis revealed that adults aged 65 and older had the highest mortality rate (17.902 per 100,000). Regarding racial disparities, white people had significantly higher mortality rates (5.996 per 100,000) compared to Black or African American(1.291 per 100,000) and Hispanic or Latino(1.018 per 100,000). Geographically, the Southern region had the highest mortality rate (5.452 per 100,000), and rural areas (5.960 per 100,000) had higher rates than metropolitan areas (5.160 per 100,000). State-level analysis showed that Idaho, Oklahoma, West Virginia, Kentucky, and Delaware ranked highest in mortality rates nationwide.

**Conclusion:**

Skin malignant neoplasm mortality in the United States shows an overall declining trend, but significant disparities exist across demographic characteristics and geographic regions. These findings provide important evidence for developing targeted prevention strategies and resource allocation for high-risk populations. Future research should further explore potential factors behind these disparities, including socioeconomic factors, healthcare accessibility, and environmental exposures.

## 1 Introduction

Skin malignant neoplasms are common malignancies with increasing global incidence, primarily including malignant melanoma (MM) and non-melanoma skin cancer (NMSC) (1). According to the World Health Organization, approximately 3-4 million new skin cancer cases are diagnosed globally each year, with MM accounting for only about 5% of all skin cancers but causing approximately 80% of skin cancer-related deaths (2). In the United States, skin cancer is the most common type of malignancy, with over 5 million newly diagnosed cases annually, representing more than 40% of all new cancer cases, and the incidence continues to rise (3). According to the American Cancer Society, an estimated 99,780 new MM cases were expected in the United States in 2022, resulting in approximately 7,650 deaths, while new cases of NMSC, including basal cell carcinoma and squamous cell carcinoma, were estimated to exceed 5 million (4).

However, it is important to acknowledge that death certificate data has well-documented limitations in accurately capturing NMSC-related mortality. NMSC deaths are frequently underreported on death certificates due to their generally lower case-fatality rates compared to melanoma, potential miscoding as other causes of death, and insufficient recognition by certifying physicians of NMSC’s contribution to mortality in patients with multiple comorbidities (5, 6). Additionally, the identification and coding of NMSC as the underlying cause of death may be inconsistent across healthcare settings and geographic regions due to variations in medical training, autopsy practices, and awareness of skin cancer’s lethal potential (7). These limitations mean that mortality statistics derived from death certificate data may systematically underestimate the true burden of NMSC-related deaths and may not fully capture the epidemiological patterns specific to different NMSC subtypes such as squamous cell carcinoma and basal cell carcinoma (8).

Ultraviolet (UV) radiation is recognized as the primary environmental risk factor for skin malignant neoplasms, with cumulative long-term UV exposure closely associated with NMSC development, while intermittent intense UV exposure is more strongly related to MM occurrence (5). While various environmental and behavioral factors may potentially influence population-level skin cancer rates over time, establishing direct causal relationships between specific exposures and observed mortality trends requires comprehensive exposure assessment and analytical study designs that are beyond the scope of death certificate-based surveillance studies. These data limitations have important implications for the interpretation of population-based skin cancer mortality studies. When analyzing aggregate skin malignant neoplasm mortality data that combines melanoma (ICD-10: C43) and NMSC (ICD-10: C44), the observed trends and demographic disparities may be disproportionately driven by melanoma mortality patterns due to more accurate reporting of melanoma deaths and their higher case-fatality rates. This could potentially mask important epidemiological characteristics specific to NMSC, including differences in age distribution, occupational risk factors, and response to preventive interventions. Furthermore, the underrepresentation of NMSC deaths in mortality statistics may lead to underestimation of skin cancer’s overall public health impact, particularly in populations with higher proportions of NMSC relative to melanoma, such as certain occupational groups with chronic sun exposure or elderly populations (9, 10). Understanding these limitations is crucial for appropriate interpretation of temporal trends, demographic disparities, and the evaluation of prevention and treatment interventions in population-based skin cancer mortality surveillance. Other risk factors include fair skin, multiple nevi, family history, immunosuppression, and specific gene mutations (11). In recent decades, the disease burden of skin cancer has increased globally due to climate change, ozone depletion, population aging, and increased outdoor recreational activities (12). Simultaneously, the incidence and mortality of skin malignant neoplasms exhibit significant geographical and demographic disparities, associated with factors such as UV exposure levels, skin type distribution, prevention awareness, and healthcare accessibility (13).

In the United States, the epidemiological characteristics of skin malignant neoplasms show significant racial/ethnic disparities. The MM incidence rate among white people is approximately 20 times higher than that among black people, with Hispanic populations falling between these two groups (14). However, non-white people racial groups are often diagnosed at later stages and have worse prognoses (15). Furthermore, gender, age, socioeconomic status, and geographic region are also important factors influencing skin cancer incidence and mortality (16, 17). Despite significant advances in skin cancer prevention and treatment in the United States over the past few decades, including public health campaigns, early screening, and the introduction of targeted and immunotherapies, the long-term trends and influencing factors of skin cancer-related mortality have not been comprehensively evaluated (18, 19).

In recent years, several studies have analyzed skin cancer mortality trends in specific regions or populations in the United States, but most are limited to short-term observations or specific subgroups (20). Guy et al. studied changes in US MM mortality from 2001 to 2010, finding that despite an overall increase in incidence, mortality rates remained relatively stable (21). Jemal et al. analyzed trends in melanoma incidence and mortality in the United States from 1992 to 2006, noting that mortality rates continued to rise among white people males while remaining stable or declining in other populations (16). However, most of these studies employed traditional linear regression methods, which cannot accurately identify turning points and change patterns in long-term trends. Additionally, few studies have simultaneously examined the impact of multidimensional factors such as race/ethnicity, age, gender, geographic region, and rural-urban differences on skin cancer mortality (22, 23).

Joinpoint regression analysis is an effective method for evaluating changes in long-term health indicator trends, capable of precisely identifying turning points in indicator changes and differences in trends before and after these points and has been widely applied in cancer epidemiological research (24). The Wide-ranging Online Data for Epidemiologic Research (WONDER) database maintained by the Centers for Disease Control and Prevention (CDC) provides detailed cause-of-death statistics nationwide, offering a valuable resource for comprehensively assessing trends in skin malignant neoplasm mortality (25). Analyses based on this database can reveal long-term patterns of mortality changes, evaluate the effectiveness of prevention and treatment interventions, and provide evidence for developing targeted public health strategies (26).

With the development of precision medicine and public health precision intervention concepts, understanding the detailed patterns of skin malignant neoplasm mortality has become particularly important (27). Analyzing differences between subgroups and their changing trends helps identify high-risk populations, optimize resource allocation, and evaluate the implementation effects of existing interventions (28). Based on this background, this study aims to systematically analyze the temporal trends of skin malignant neoplasm mortality among US adults from 1999 to 2020 and its characteristics across dimensions such as gender, age, race/ethnicity, geographic region, and rural-urban differences using the CDC WONDER database and Joinpoint regression method. It is important to note that this mortality surveillance study focuses on describing temporal patterns and demographic disparities rather than establishing causal relationships with specific environmental exposures, risk factors, or interventions. While we may observe temporal associations between mortality trends and various contemporaneous developments, our ecological study design using aggregate death certificate data does not permit definitive causal inferences about the underlying factors driving these observed patterns. We analyzed skin malignant neoplasms in aggregate (combining ICD-10 codes C43 for melanoma and C44 for non-melanoma skin cancers) rather than conducting separate subtype-specific analyses for several reasons (1): the CDC WONDER standard query system does not permit separate tabulation of individual ICD-10 subcodes within the broader skin malignant neoplasm category; (2) death certificate data may be subject to coding misclassification between melanoma and non-melanoma subtypes; (3) melanoma accounts for approximately 80% of all skin cancer deaths, meaning our aggregate trends predominantly reflect melanoma mortality patterns; and (4) this approach is consistent with established population-based skin cancer surveillance methodologies and facilitates comparison with existing literature (1, 2). While this aggregate approach cannot capture subtype-specific epidemiological patterns, it provides valuable insights for comprehensive skin cancer prevention and control policies. This will provide comprehensive and reliable epidemiological evidence for skin cancer prevention and control, as well as reference for rational allocation of health resources.

## 2 Materials and methods

### 2.1 Data sources

This study utilized the Wide-ranging Online Data for Epidemiologic Research (WONDER) database from the Centers for Disease Control and Prevention (CDC) (https://wonder.cdc.gov/) to obtain skin malignant neoplasm mortality data for US adults (≥25 years) from 1999 to 2020.We focused on adults aged 25 years and older because skin malignant neoplasm mortality is extremely rare in younger populations, which could result in unstable rate estimates and potential data suppression issues in the CDC WONDER database. Additionally, this age group represents the primary target population for skin cancer prevention and control policies and aligns with the majority of population-based skin cancer surveillance studies. According to the International Classification of Diseases, 10th Revision (ICD-10), skin malignant neoplasms include malignant melanoma (C43) and other malignant neoplasms of skin (C44).For this study, we analyzed skin malignant neoplasms in aggregate by combining ICD-10 codes C43 (melanoma) and C44 (non-melanoma skin cancers) rather than conducting separate subtype-specific analyses. This approach was adopted for several reasons: (1) the CDC WONDER standard query system does not permit separate tabulation of individual ICD-10 subcodes when requesting detailed demographic stratifications; (2) death certificate data may be subject to coding misclassification between melanoma and NMSC subtypes; (3) melanoma accounts for approximately 80% of skin cancer deaths, meaning our aggregate trends predominantly reflect melanoma mortality patterns; and (4) this methodology is consistent with established population-based skin cancer surveillance approaches. While this aggregate approach cannot capture subtype-specific epidemiological patterns, it provides valuable insights for comprehensive skin cancer mortality surveillance and facilitates comparison with existing literature. Information extracted from the database included year of death, number of deaths, crude mortality rate, age-adjusted mortality rate, gender, age group, race/ethnicity, and geographic region. The mortality data in the CDC WONDER database is based on death certificates from the National Vital Statistics System, covering all 50 US states and the District of Columbia. Age-adjusted mortality rates were calculated using the 2000 US standard population as the reference, expressed per 100,000 population, which eliminates the effect of changes in population age structure on mortality rates across different time periods (29).

### 2.2 Statistical analysis

Joinpoint regression analysis was employed to evaluate the temporal trends of skin malignant neoplasm mortality. Joinpoint regression identifies significant turning points (joinpoints) in data trends and estimates the rate of change within each linear segment. We used the Joinpoint Regression Program (version 4.9.0) developed by the National Cancer Institute for the analysis. The optimal number of joinpoints for each analysis was determined using the software’s built-in model selection procedure, which employs Monte Carlo permutation methods to test the statistical significance of each potential joinpoint. We set the maximum number of joinpoints to 5, which is appropriate for our 22-year study period. The model selection process begins with a straight line (no joinpoints) and sequentially tests models with increasing numbers of joinpoints until additional joinpoints no longer provide statistically significant improvements in model fit (P≥0.05). The final model selected was the one with the maximum number of statistically significant joinpoints, ensuring optimal detection of trend changes while maintaining model parsimony.

For each analysis, we calculated the annual percent change (APC) and its 95% confidence interval (95% CI). The APC represents the annual relative percentage change in mortality rates during a specific time period. Additionally, we calculated the average annual percent change (AAPC) for the entire study period (1999–2020), which is a weighted average of multiple APC values. An APC or AAPC value greater than 0 with statistical significance (P<0.05) indicates a significant increase in mortality rate, a value less than 0 with statistical significance indicates a significant decrease, while values without statistical significance indicate stable mortality rates.

We conducted stratified trend analyses according to the following variables (1): gender (male, female) (2); age group (25-44 years, 45-64 years, 65+ years) (3); race/ethnicity (Hispanic or Latino, Black or African American, and White people) (30) (4); geographic region (Northeast, Midwest, South, West) (5); counties classified as urban (large metropolitan areas, medium/small metropolitan areas) and rural (population <50,000) according to the 2013 US Census classification. Data suppression in the CDC WONDER database occurs when mortality counts are fewer than 10 deaths to protect confidentiality and ensure statistical reliability. For stratified analyses, we excluded subgroups and time periods where data were suppressed or deemed unreliable by CDC WONDER. Additionally, when mortality counts were between 10-20 deaths (which could result in unstable rate estimates with wide confidence intervals), we interpreted these results with appropriate caution and clearly noted the potential for statistical instability in our findings. For racial/ethnic subgroups, particularly Black or African American and Hispanic or Latino populations that had relatively low skin cancer mortality counts, we focused our analysis on overall trends rather than detailed temporal pattern analysis when sample sizes were insufficient for reliable joinpoint regression modeling. All reported rates and trends were based only on non-suppressed data that met CDC WONDER’s standards for statistical reliability. All statistical tests were two-sided, with P<0.05 considered statistically significant. Except for the Joinpoint regression analysis, other statistical analyses was R software (version 4.3.0, R Foundation for Statistical Computing, Vienna, Austria).

## 3 Results

### 3.1 Overall mortality characteristics and trends

**Figure 1:**
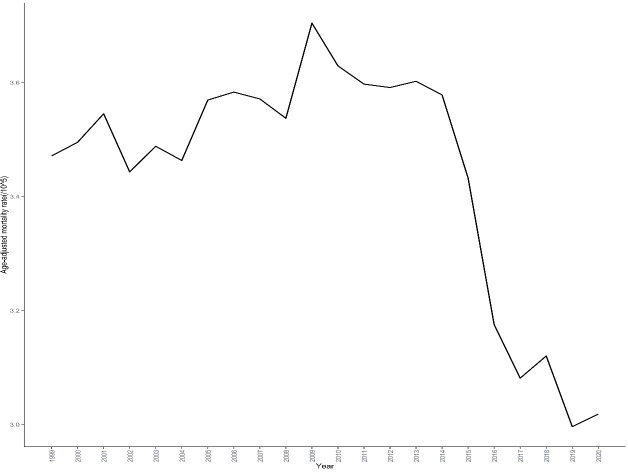
Trends in skin malignant neoplasm mortality rates in the United States, 1999-2020. This figure illustrates the temporal trends in age-adjusted mortality rates of skin malignant neoplasms over a 22-year period based on CDC WONDER database. The x-axis represents the calendar years (1999-2020), and the y-axis represents the age-adjusted mortality rate (per 100,000 population). The figure demonstrates the overall trends and fluctuations in skin malignant neoplasm mortality during the study period.

Between 1999 and 2020, a total of 251,848 skin malignant neoplasm deaths were reported in the United States, with an age-standardized mortality rate of 5.299 per 100,000 person-years (**Table 1**, **Supplementary Table 1**). Among these deaths, 167,619 were males, accounting for 66.56% of the total, while 84,229 were females, accounting for 33.44%. The age-standardized mortality rate for males (8.137 per 100,000) was significantly higher than for females (3.157 per 100,000), with a male-to-female ratio of 1.99:1. Joinpoint regression analysis results showed that the age-standardized mortality rate of skin malignant neoplasms in the United States from 1999 to 2020 exhibited an overall declining trend, but with notable turning points (**Figure 1**). The mortality rate showed a steady increasing trend from 1999 to 2014 (APC =+ 0.271%, 95% CI: 0.09 to 0.494, P = 0.018), followed by a significant decreasing trend from 2014 to 2017 (APC=-5.292%, 95% CI: -6.221 to -3.304, P = 0.027),and remained stable from 2017 to 2020 (APC=-0.563%, 95% CI: -2.256 to 2.132, P = 0.555).The average annual percent change for the entire study period (1999-2020) was -0.662% (95% CI: -0.832 to -0.501, P<0.001) (**Table 2**).

**Table 1:** Demographic distribution of skin malignant neoplasm mortality in the United States, 1999-2020.

Variables	Deaths	Population	AAMR(95%CI)
Overall	251848	4473854489	5.299(5.278-5.320)
Sex			
Male	167619	2154556911	8.137(8.097-8.177)
Female	84229	2319297578	3.157(3.135-3.179)
Race/Ethnicity			
Hispanic or Latino	2346	295429555	1.018(0.975-1.060)
Black or African American	6176	546447527	1.291(1.258-1.324)
White People	243326	3631977407	5.996(5.972-6.020)
Census Region			
Northeast	45437	827193779	4.930(4.884-4.976)
Midwest	55726	969567311	5.224(5.180-5.268)
South	95041	1652256217	5.452(5.418-5.487)
West	55644	1024837182	5.393(5.348-5.438)
Urbaniztion			
Metropolitan(Urban)			
Nonmetropolitan(Rural)	48458	678634169	5.960(5.907-6.014)
Ten-Year Age Groups	203390	3795213822	5.160(5.137-5.182)
25-44 years	14809	1851376757	0.800(0.787-0.813)
45-64 years	72492	1694001067	4.279(4.248-4.310)
65+ years	164547	928476665	17.902(17.816-17.989)

AAMR, age‐adjusted mortality rate; CI, confidence interval. This table presents the total number of deaths, population size, and age-adjusted mortality rates (per 100,000 population) with 95% confidence intervals for skin malignant neoplasms during 1999-2020, stratified by multiple demographic characteristics based on CDC WONDER database. The table includes: overall statistics, sex (male, female), race/ethnicity (Hispanic or Latino, Black or African American, White people), census region (Northeast, Midwest, South, West), urbanization level (metropolitan/urban, nonmetropolitan/rural), and ten-year age groups (25-44 years, 45-64 years, 65+ years). A total of 251,848 deaths were recorded over the 22-year period with a population base of 4,473,854,489 person-years, yielding an overall age-adjusted mortality rate of 5.299 (95% CI: 5.278-5.320). The table reveals substantial disparities in skin malignant neoplasm mortality across sex, race, region, urbanization, and age dimensions.

**Table 2:** Joinpoint regression analysis of skin malignant neoplasm mortality trends in the United States, 1999-2020.

Variables	Trend segment	Year interval	APC (95% CI)	AAPC (95% CI)	*P-*value
Entire Cohort	–	1999-2020	–	-0.662(-0.832 to -0.501)	<0.001
	1	1999-2014	0.271(0.090 to 0.494)	–	0.018
	2	2014-2017	-5.292(-6.221 to -3.304)	–	0.027
	3	2017-2020	-0.563(-2.256 to 2.132)	–	0.555
Sex					
Male	–	1999-2020	–	-0.749(-0.904 to -0.585)	<0.001
	1	1999-2013	0.604(0.356 to 0.89)	–	<0.001
	2	2013-2020	-3.400(-4.110 to -2.780)	–	<0.001
Female	–	1999-2020	–	-1.089(-1.364 to -0.807)	<0.001
	1	1999-2012	-0.334(-0.716 to 0.377)	–	0.258
	2	2012-2020	-2.304(-3.703 to -1.546)	–	<0.001
Age					
25-44	–	1999-2020	–	0.252(-0.100 to 0.692)	0.106
	1	1999-2013	0.769(0.404-3.285)	–	0.004
	2	2013-2020	-0.773(-4.089 to 0.148)	–	0.116
45-64	–	1999-2020	–	-0.770(-1.046 to -0.504)	0.003
	1	1999-2013	-0.013(-0.337 to 0.535)	–	0.955
	2	2013-2020	-2.265(-3.781 to -1.411)	–	<0.001
65+	–	1999-2020	–	0.094(-0.083 to 0.282)	0.245
	1	1999-2013	1.319(1.038 to 1.658)	–	<0.001
	2	2013-2020	-2.313(-3.126 to -1.643)	–	<0.001
Race/Ethnicity					
Hispanic or Latino	–	1999-2020	–	-0.829(-1.691 to 0.333)	0.166
Black or African American	–	1999-2020	–	-1.899(-2.335 to -1.433)	<0.001
White People	–	1999-2020	–	-0.456(-0.698 to -0.262)	<0.001
	1	1999-2014	0.521(0.172 to 0.824)	–	0.038
	2	2014-2017	-5.083(-6.199 to 0.834)	–	0.093
	3	2017-2020	-0.580(-2.934 to 2.542)	–	0.574
Census Region					
Northeast	–	1999-2020	–	-1.063(-1.309 to -0.814)	<0.001
	1	1999-2012	0.132(-0.262 to 0.673)	–	0.475
	2	2012-2020	-2.973(-4.054 to -2.198)	–	<0.001
Midwest	–	1999-2020	–	-0.325(-0.663 to 0.015)	0.062
	1	1999-2013	0.757(0.324 to 1.438)	–	0.001
	2	2013-2020	-2.455(-4.294 to -1.348)	–	<0.001
South	–	1999-2020	–	-0.873(-1.041 to -0.715)	<0.001
	1	1999-2014	0.085(-0.132 to 0.331)	–	0.328
	2	2014-2017	-5.198(-6.094 to -0.016)	–	0.049
	3	2017-2020	-1.216(-3.146 to 1.203)	–	0.283
West	–	1999-2020	–	-0.640(-0.855 to -0.400)	<0.001
	1	1999-2011	0.767(0.323 to 1.358)	–	0.002
	2	2011-2020	-2.486(-3.246 to -1.868)	–	<0.001
Urbaniztion					
Metropolitan(Urban)	–	1999-2020	–	-0.779(-0.981 to -0.598)	<0.001
	1	1999-2014	0.237(0.020 to 0.501)	–	0.042
	2	2014-2017	-5.661(-6.702 to -0.098)	–	0.046
	3	2017-2020	-0.829(-3.078 to 2.082)	–	0.461
Nonmetropolitan(Rural)	–	1999-2020	–	-0.243(-0.599 to 0.069)	0.103
	1	1999-2014	0.599(0.246 to 1.171)	–	0.002
	2	2014-2020	-2.318(-4.658 to -1.153)	–	<0.001

This table presents the results of joinpoint regression analysis examining temporal trends in skin malignant neoplasm mortality during 1999-2020, stratified by multiple demographic characteristics based on CDC WONDER database. The table includes: trend segment number, year interval, annual percent change (APC) with 95% confidence intervals, average annual percent change (AAPC) with 95% confidence intervals, and P-values. The analysis encompasses overall population, sex (male, female), age groups (25-44 years, 45-64 years, 65+ years), race/ethnicity (Hispanic or Latino, Black or African American, White people), census region (Northeast, Midwest, South, West), and urbanization level (metropolitan/urban, nonmetropolitan/rural). Results show an overall AAPC of -0.662 (95% CI: -0.832 to -0.501, P<0.001), indicating a significant declining trend in mortality over the 22-year period, though the timing of inflection points and rates of decline varied across demographic subgroups.

### 3.2 Gender difference analysis

**Figure 2:**
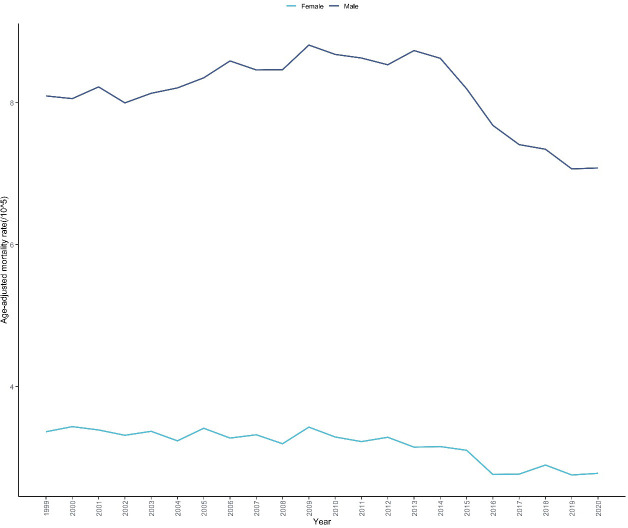
Trends in skin malignant neoplasm mortality rates by sex in the United States, 1999-2020. This figure illustrates the temporal trends in age-adjusted mortality rates of skin malignant neoplasms stratified by sex over a 22-year period based on CDC WONDER database. The x-axis represents the calendar years (1999-2020), and the y-axis represents the age-adjusted mortality rate (per 100,000 population). Two trend lines are displayed, representing males and females respectively. The figure enables visual comparison of mortality disparities between sexes and their respective temporal patterns.

Throughout the study period, the mortality rate of skin malignant neoplasms in males consistently exceeded that of females (**Table 1**, **Supplementary Table 1**). In 2020, the AAMRs for males and females were 7.079 per 100,000 (95% CI: 6.924-7.234) and 2.777 per 100,000 (95% CI: 2.690-2.865), respectively. The trends in skin malignant neoplasm mortality rates also showed notable differences between males and females (**Figure 2**). Male mortality rates exhibited an increasing trend from 1999 to 2013 (APC =+ 0.604%, 95% CI: 0.356 to 0.889, P<0.001), followed by a significant decreasing trend from 2013 to 2020 (APC=-3.400%, 95% CI: -4.110 to -2.780, P<0.001).Female mortality rates remained relatively stable from 1999 to 2012 (APC=-0.334%, 95% CI: -0.716 to 0.377, P = 0.258),followed by a significant decreasing trend from 2012 to 2020 (APC=-2.304%, 95% CI: -3.703 to -1.546, P<0.001). For the entire study period, the AAPC for males was -0.749% (95% CI: -0.904 to -0.585, P<0.001), higher than the AAPC for females (-1.089%, 95% CI: -1.364 to -0.807, P<0.001) (**Table 2**).

### 3.3 Age group difference analysis

**Figure 3:**
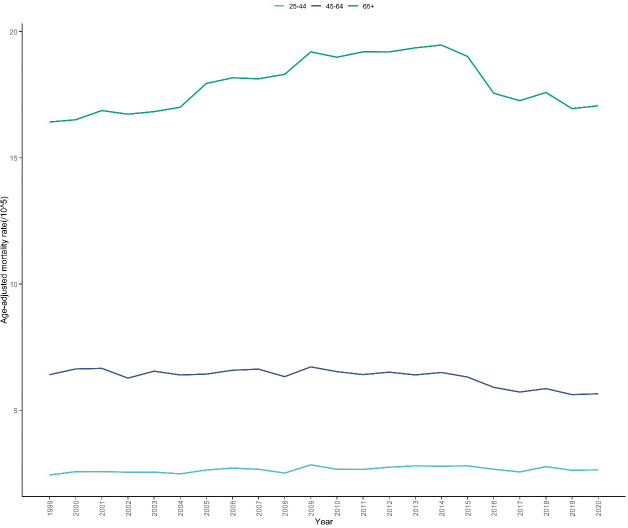
Trends in skin malignant neoplasm mortality rates by age groups in the United States, 1999-2020. This figure illustrates the temporal trends in age-adjusted mortality rates of skin malignant neoplasms stratified by age groups over a 22-year period based on CDC WONDER database. The x-axis represents the calendar years (1999-2020), and the y-axis represents the age-adjusted mortality rate (per 100,000 population). Three trend lines are displayed, representing different age groups: 25-44 years,45-64 years, and ≥65 years. The figure enables visual comparison of mortality disparities across age groups and their respective temporal patterns.

Significant differences were observed in skin malignant neoplasm mortality rates and their trends across different age groups (**Table 1**, **Supplementary Table 2**). Mortality rates increased in a stepwise manner with age, with the mortality rate in the 65+ age group (17.902 per 100,000) being 22.38 times higher than that in the 25-44 age group (0.800 per 100,000). Joinpoint regression analysis (**Figure 3**) showed that the mortality rate in the 25-44 age group exhibited a significant increasing trend from 1999 to 2013 (APC =+ 0.769%, 95% CI: 0.404 to 3.285, P = 0.004),followed by a stable period from 2013 to 2020 (APC=-0.773%, 95% CI: -4.089 to 0.148, P = 0.116);the mortality rate in the 45-64 age group showed an overall declining trend with a turning point in 2013,remaining stable from 1999 to 2013 (APC=-0.013%, 95% CI: -0.337 to 0.535, P = 0.955) and a significant decreasing trend from 2013 to 2020 (APC=-2.265%, 95% CI: -3.781 to -1.411, P<0.001); while the mortality rate in the 65+ age group had an APC of +1.319% (95% CI: 1.038 to 1.658, P<0.001) from 1999 to 2013, followed by a significant decreasing trend from 2013 to 2020 (APC=-2.313%, 95% CI: -3.126 to -1.643, P<0.001). For the entire study period, the AAPC for the 25-44 age group was +0.252% (95% CI: -0.100 to 0.692, P = 0.106); the AAPC for the 45-64 age group was -0.770% (95% CI: -1.046 to -0.504, P = 0.003); and the AAPC for the 65+ age group showed stability at +0.094% (95% CI: -0.083 to 0.282, P = 0.245) (**Table 2**).

### 3.4 Racial/ethnic difference analysis

**Figure 4:**
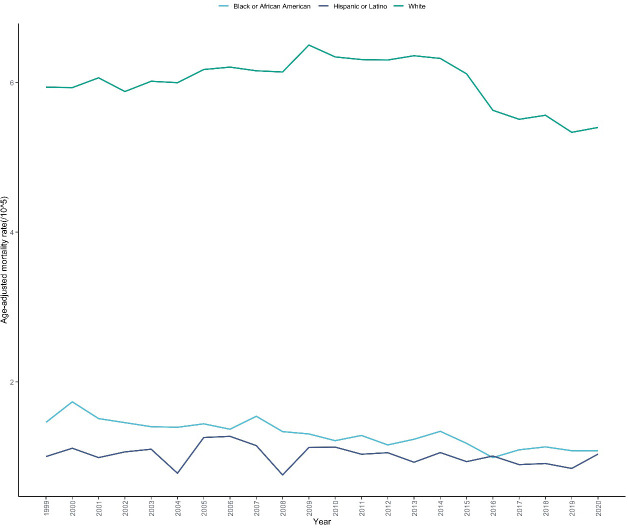
Trends in skin malignant neoplasm mortality rates by race/ethnicity in the United States, 1999-2020. This figure illustrates the temporal trends in age-adjusted mortality rates of skin malignant neoplasms stratified by race/ethnicity over a 22-year period based on CDC WONDER database. The x-axis represents the calendar years (1999-2020), and the y-axis represents the age-adjusted mortality rate (per 100,000 population).Three trend lines are displayed, representing Hispanic or Latino, Black or African American and White populations respectively. The figure enables visual comparison of mortality disparities between racial/ethnic groups and their respective temporal patterns.

Skin malignant neoplasm mortality rates differed significantly across racial/ethnic groups (**Table 1**, **Supplementary Table 3**). White people had the highest mortality rate (5.996 per 100,000), followed by Black or African American (1.291 per 100,000), while Hispanic or Latino had the lowest mortality rate (1.018 per 100,000). Trends in mortality rates also varied across racial/ethnic groups (**Figure 4**). Among White people, the mortality rate increased significantly from 1999 to 2014 (APC =+ 0.521%, 95% CI: 0.172 to 0.824, P = 0.038),followed by a non-significant decline from 2014 to 2017 (APC=-5.083%, 95% CI: -6.199 to 0.834, P = 0.093), and remained stable from 2017 to 2020 (APC=-0.580%, 95% CI: -2.934 to 2.542, P = 0.574);mortality rates for Black or African American and Hispanic or Latino remained relatively stable throughout the entire study period, with AAPCs of -1.899% (95% CI: -2.335 to -1.433, P<0.001) and -0.829% (95% CI: -1.691 to 0.333, P = 0.166), respectively; for the entire study period, the AAPC for White people were -0.456% (95% CI: -0.698 to -0.262, P<0.001) (**Table 2**).

### 3.5 Geographical region difference analysis

**Figure 5:**
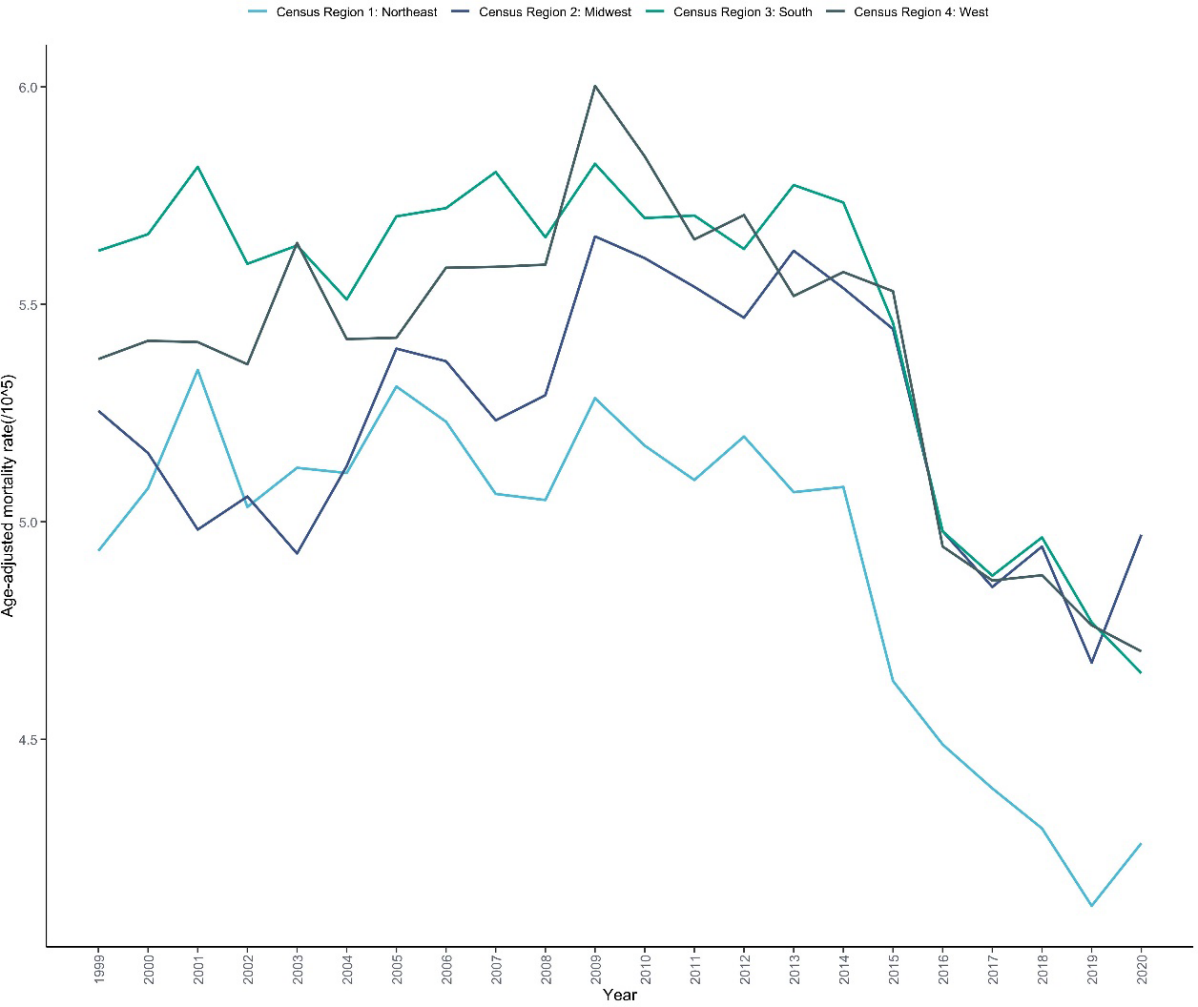
Trends in skin malignant neoplasm mortality rates by census region in the United States, 1999-2020. This figure illustrates the temporal trends in age-adjusted mortality rates of skin malignant neoplasms stratified by census region over a 22-year period based on CDC WONDER database. The x-axis represents the calendar years (1999-2020), and the y-axis represents the age-adjusted mortality rate (per 100,000 population). Four trend lines are displayed, representing the four census regions: Northeast, Midwest, South, and West. The figure enables visual comparison of mortality disparities across geographic regions and their respective temporal patterns.

Significant differences in skin malignant neoplasm mortality rates were also observed across the four major geographic regions of the United States (**Table 1**, **Supplementary Table 4**). The South had the highest mortality rate (5.452 per 100,000), followed by the West (5.393 per 100,000), Midwest (5.224 per 100,000), and Northeast (4.930 per 100,000). Joinpoint regression analysis revealed varying trends in mortality rates across the four regions (**Figure 5**). The Southern region remained stable from 1999 to 2014 (APC =+ 0.085%, 95% CI: -0.132 to 0.331, P = 0.328), followed by a declining trend, with a rapid decrease from 2014 to 2017 (APC=-5.198%, 95% CI: -6.094 to -0.016, P = 0.049), and remained stable from 2017 to 2020 (APC=-1.216%, 95% CI: -3.146 to 1.203, P = 0.283); the Western region exhibited a significant decreasing trend after 2014 (APC=-2.486%, 95% CI: -3.246 to -1.868, P<0.001); both the Midwest and Northeast regions showed trends of initial increase followed by decrease, with turning points in 2013 and 2012, respectively; for the entire study period, the AAPC for the Southern region was -0.873% (95% CI: -1.041 to -0.715, P<0.001), for the Western region was -0.640% (95% CI: -0.855 to -0.400, P<0.001), and for the Midwest and Northeast regions were -0.325% (95% CI: -0.663 to 0.015, P = 0.062) and -1.063% (95% CI: -1.309 to -0.814, P<0.001), respectively (**Table 2**).

### 3.6 Urban and rural differences

**Figure 6:**
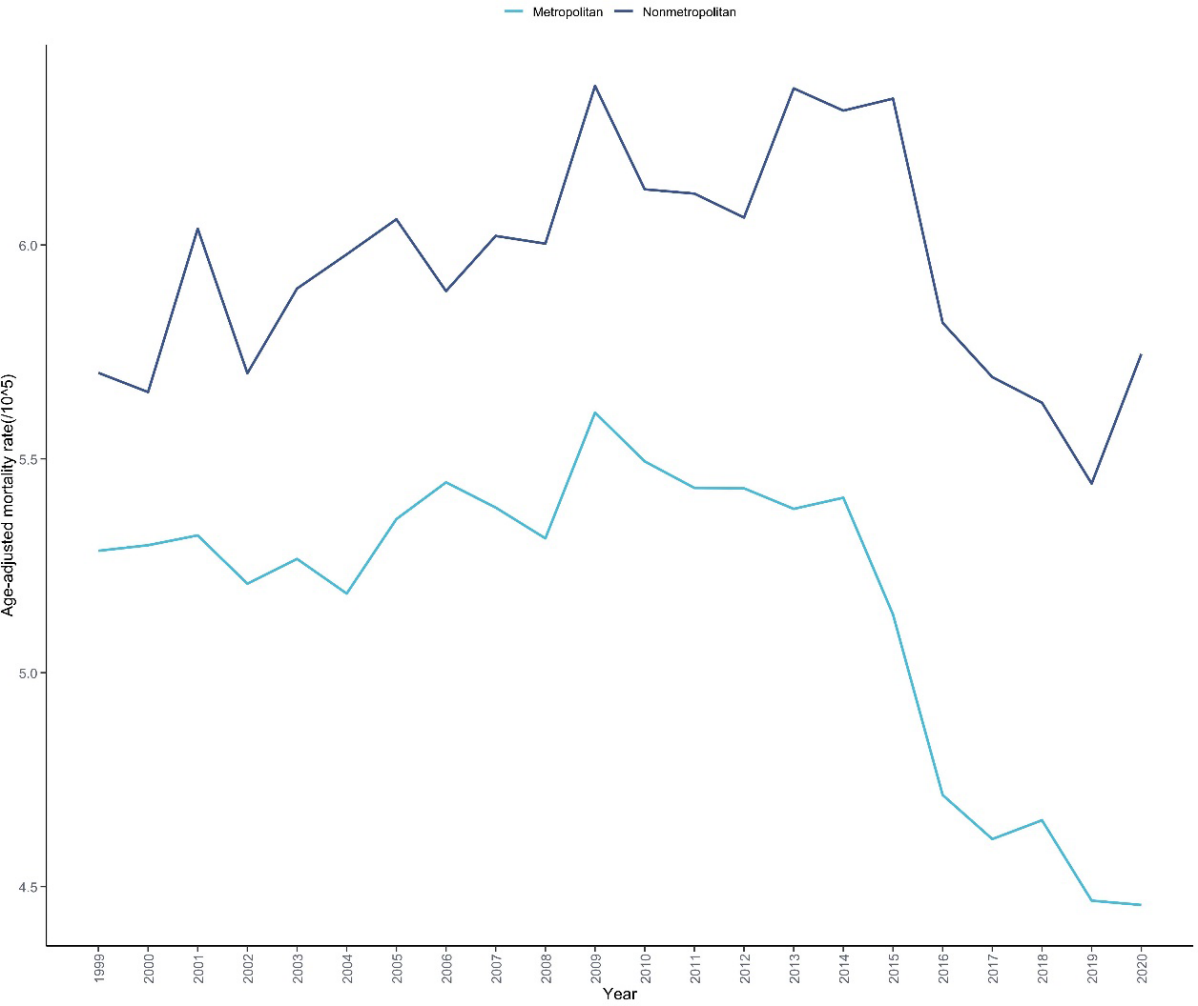
Trends in skin malignant neoplasm mortality rates by urbanization level in the United States, 1999-2020. This figure illustrates the temporal trends in age-adjusted mortality rates of skin malignant neoplasms stratified by urbanization level over a 22-year period based on CDC WONDER database. The x-axis represents the calendar years (1999-2020), and the y-axis represents the age-adjusted mortality rate (per 100,000 population). Two trend lines are displayed, representing metropolitan and nonmetropolitan areas respectively. The figure enables visual comparison of mortality disparities between urban and rural areas and their respective temporal patterns.

Urban-rural analysis showed (**Table 1**, **Supplementary Table 5**) that in 2020, rural areas had the highest mortality rate of skin malignant neoplasms at 5.960 per 100,000 (95% CI: 5.907-6.014), significantly higher than the rate of 5.160 per 100,000 (95% CI: 5.137-5.182) in metropolitan areas. Joinpoint analysis (**Figure 6**) revealed that mortality rates in metropolitan areas showed an overall declining trend (AAPC=-0.779%, 95% CI: -0.981 to -0.598, P<0.001), with a slow increasing trend from 1999 to 2014 (APC =+ 0.237%, 95% CI: 0.02 to 0.501, P = 0.042), a rapid declining trend from 2014 to 2017 (APC=-5.661%, 95% CI: -6.702 to -0.098, P = 0.046),and remained stable from 2017 to 2020 (APC=-0.829%, 95% CI: -3.078 to 2.082, P = 0.461);mortality rates among residents in non-metropolitan areas showed an overall declining trend (AAPC=-0.243%, 95% CI: -0.599 to 0.069, P = 0.103), but exhibited an increasing trend from 1999 to 2014 (APC =+ 0.599%, 95% CI: 0.246 to 1.171, P = 0.002), followed by a rapid declining trend from 2014 to 2020 (APC=-2.318%, 95% CI: -4.658 to -1.153, P<0.001) (**Table 2**).

### 3.7 Trends stratified by state

**Figure 7:**
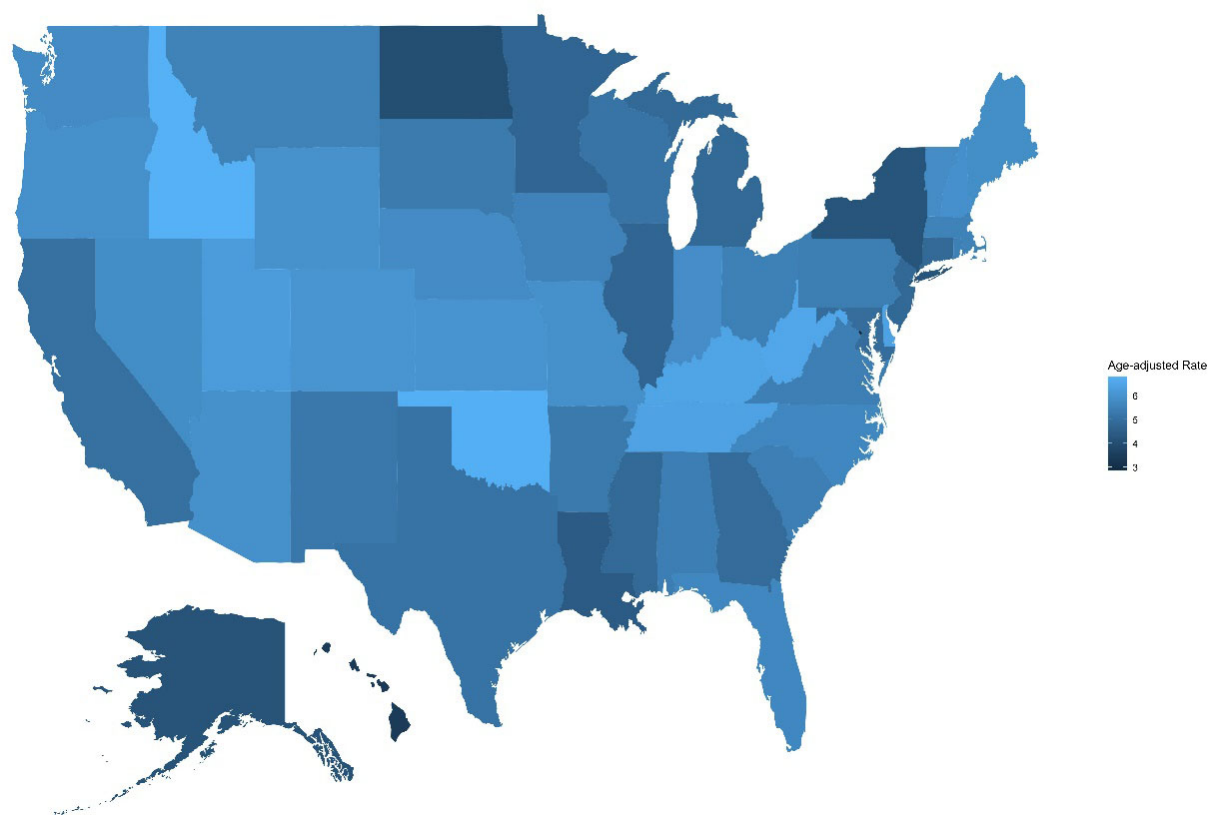
Geographic distribution of age-adjusted mortality rates (AAMR) of skin malignant neoplasms by state in the United States, 1999-2020. This figure presents a choropleth map displaying the geographic distribution of age-adjusted mortality rates (AAMR) for skin malignant neoplasms across U.S. states during 1999-2020, based on CDC WONDER database. The map uses a color gradient to represent varying mortality levels across states, with color intensity indicating the magnitude of mortality rates. The figure facilitates visual identification of geographic hotspots, distribution patterns of high-burden and low-burden states, and potential geographic clustering of skin malignant neoplasm mortality. The legend indicates the AAMR value ranges and their corresponding color classifications.

State-level analysis revealed considerable heterogeneity. Idaho had the highest AAMR at 6.792 per 100,000 (95% CI: 6.449-7.134), followed by Oklahoma with an AAMR of 6.742 per 100,000 (95% CI: 6.528-6.955), West Virginia (6.511 per 100,000; 95% CI: 6.229-6.794), Kentucky (6.467 per 100,000; 95% CI: 6.272-6.661), and Delaware (6.421 per 100,000; 95% CI: 6.007-6.836). In contrast, the District of Columbia had the lowest AAMR (2.848 per 100,000; 95% CI: 2.487-3.208), followed by Hawaii (3.389 per 100,000; 95% CI: 3.149-3.630), and North Dakota (3.988 per 100,000; 95% CI: 3.617-4.358).Higher mortality states predominantly located in the Western, South-Central, and Appalachian regions, while lower mortality states are mainly concentrated in densely populated Northeastern areas. (**Supplementary Table 6**, **Figure 7**).

## 4 Discussion

This study, based on the CDC WONDER database, systematically analyzed the long-term trends and disparities in skin malignant neoplasm mortality rates among American adults from 1999 to 2020. The findings revealed distinct phasic changes in U.S. skin malignant neoplasm mortality rates over these 22 years: a steady increase from 1999 to 2014, a significant decrease from 2014 to 2017, and a stable declining trend from 2017 to 2020. This pattern of initial increase followed by decrease generally aligns with skin cancer mortality trends reported in multinational studies (31, 32).

The turning point in mortality rates observed in 2014 holds significant public health implications. The observed temporal decline beginning in 2014 coincides chronologically with multiple developments in healthcare policy and cancer treatment; however, the ecological nature of this analysis requires that we emphasize important limitations in interpreting these patterns. Specifically, during this period several developments occurred that theoretically could have influenced melanoma mortality: the Affordable Care Act implementation (begun in 2010) expanded health insurance coverage (33); the FDA approved vemurafenib for advanced melanoma in 2011, followed by approvals of immune checkpoint inhibitors (ipilimumab in 2011, pembrolizumab and nivolumab between 2014-2015), which demonstrated clinical efficacy in clinical trials; and the U.S. Department of Health issued ‘The Surgeon General’s Call to Action to Prevent Skin Cancer’ in 2014, which may have increased public awareness of skin cancer prevention (34). However, this ecological study design using aggregate mortality data does NOT permit us to establish whether any of these developments directly caused the observed mortality decline. This is a critical limitation: we can observe the temporal sequence of events (policy changes, therapeutic approvals, and mortality changes), but we cannot infer causation from temporal correlation. Multiple unmeasured and confounding factors at population level—including changes in screening practices, shifts in disease stage at diagnosis, coding practices in death certificates, population-level behavioral changes in sun exposure, or healthcare-seeking behaviors could independently or collectively explain part or all of the observed trend, entirely independent of the specific policy or therapeutic developments mentioned above. The mortality decline we observe may reflect one, several, all, or none of the contemporaneous interventions; additional individual-level data with clinical detail would be required to distinguish between these possibilities (35). We emphasize this limitation because mortality surveillance studies like ours are descriptive rather than etiologic: they can identify that changes have occurred and when they occurred, but they cannot reliably identify why changes occurred without individual-level exposure data and adjustment for confounding factors that are not available in aggregate death certificate data.

Gender disparity analysis showed that skin malignant neoplasm mortality rates in males consistently exceeded those in females (8.137 vs. 3.157 per 100,000), which is consistent with previous studies (36). This disparity may stem from multiple factors: on one hand, males typically spend more time in outdoor work and activities, resulting in greater cumulative UV exposure (37); on the other hand, research indicates that males have lower compliance with sun protection measures and perform skin self-examinations less frequently than females (38). Notably, the declining trend in mortality rates was weaker in males (AAPC=-0.749%) compared to females (AAPC=-1.089%). One speculative explanation, based on limited observational evidence, is that males may be diagnosed at more advanced disease stages and may demonstrate relatively delayed healthcare-seeking behavior compared to females (39). However, this interpretation is highly speculative for several reasons and cannot be directly verified in our aggregated mortality data: our data cannot distinguish the relative importance of these proposed mechanisms, nor can we exclude alternative explanations such as differential lifestyle exposures between sexes, variations in health awareness or disease perception, or potential differences in how diagnostic criteria are applied across sexes in clinical practice. Furthermore, based on limited evidence, some researchers have speculated that estrogen may have a protective effect on melanocytes. While this hypothesis could potentially contribute to explaining the lower incidence and mortality rates observed in females (40), it is important to note that this remains a speculative interpretation that cannot be directly verified in our aggregated mortality data.

Age group analysis revealed a significant increase in mortality rates with advancing age, with the mortality rate in the 65+ age group (17.902 per 100,000) being 22.38 times that of the 25-44 age group. This age gradient aligns with the cumulative UV exposure theory (41). Interestingly, different age groups exhibited distinct patterns of change: the younger population aged 25-44 showed increasing mortality rates before 2013, followed by a decline after 2013; while the elderly population aged 65+ demonstrated a more pronounced pattern of initial increase (APC =+ 1.319%) followed by decrease (APC=-2.313%) during the same period. This disparity may be associated with the differential impact of novel therapeutic approaches across different age groups. Certain clinical studies (such as research by Eguchi et al.) have reported observations suggesting that younger melanoma patients may demonstrate superior responses to immunotherapy compared to elderly patients (42). While this clinical observation, if similarly applicable in our population, could speculatively contribute to explaining part of the observed age-related mortality disparities, it is important to emphasize that such an interpretation is highly speculative for several reasons (1): clinical observations may not be fully generalizable to the population level (2); multiple other factors (such as differential comorbidity patterns across age groups, variations in disease stage at diagnosis, and differential access to treatment) may also contribute substantially to the observed age disparities (3); our ecological study design precludes direct assessment of treatment response efficacy in the actual population.

Racial/ethnic disparity is another significant finding of this study. White people had significantly higher mortality rates (5.996 per 100,000) compared to Black or African American (1.291 per 100,000) and Hispanic or Latino (1.018 per 100,000), primarily due to differences in skin pigmentation and sensitivity to ultraviolet radiation (43). Notably, despite lower mortality rates among non-White people racial groups, they are typically diagnosed at later stages and have poorer prognoses (44). This ‘paradox’ may be related to factors such as inequitable access to healthcare resources, disparities in health insurance coverage, insufficient awareness of skin cancer risk, and limited ability of healthcare professionals to identify skin cancer on darker skin (45). Research by Hu et al. indicates that although Black or African American have a lower incidence of melanoma, their 5-year relative survival rate is significantly lower than that of White people (70% vs. 94%) (46).

Geographic regional analysis revealed that the Southern United States had the highest mortality rate (5.452 per 100,000), which is associated with factors such as high ultraviolet radiation intensity, extended outdoor activity time, and relatively low skin cancer screening coverage in the region (47). However, the Southern region experienced the most significant decrease in mortality rates during 2014-2017 (APC=-5.198%), potentially reflecting the effects of focused implementation of skin cancer prevention and treatment interventions in high-risk areas (48). Research by Watson et al. suggests that awareness of skin cancer protection has increased in the Southern region in recent years, with increased use of sun protection products, which may partially explain the observed decrease in mortality rates (48).

Urban-rural disparity is another important finding of this study. Rural areas had higher mortality rates (5.960 per 100,000) than metropolitan areas (5.160 per 100,000), which is consistent with findings by Zahnd et al. (49). This disparity may reflect multiple challenges faced in rural areas, including shortages of dermatology specialists, low health insurance coverage, limited skin cancer screening programs, and barriers related to travel distance and costs for medical visits (50). Research by Brewer et al. indicates that rural residents experience an average delay of 39 days longer than urban residents in accessing dermatological specialist care, which may lead to delayed diagnosis and disease progression (51).

State-level analysis revealed significant geographic heterogeneity, with Idaho, Oklahoma, West Virginia, Kentucky, and Delaware ranking among the highest in mortality rates nationwide. These high-mortality states are distributed across different regions of the United States, indicating that skin cancer mortality risk is influenced by multiple regional factors. For example, the high mortality rates in Idaho and Oklahoma may be related to high proportions of outdoor occupations and greater intensity of ultraviolet exposure (22), while the high mortality rates in West Virginia and Kentucky may be associated with insufficient medical resources, low socioeconomic status, and low health insurance coverage (52). In contrast, the low mortality rates in the District of Columbia and Hawaii may benefit from better healthcare accessibility and higher proportions of non-White populations (53).

It is important to note that our study analyzed overall skin malignant neoplasm mortality by combining ICD-10 codes C43 (malignant melanoma) and C44 (other malignant neoplasms of skin) without distinguishing between subtypes. This aggregation has important implications for the interpretation of our findings. Melanoma and non-melanoma skin cancers (NMSC) have fundamentally different epidemiological profiles: melanoma accounts for only approximately 5% of skin cancer cases but causes 75–80% of skin cancer-related deaths, while NMSC, although far more prevalent, has substantially lower case-fatality rates (2, 4). Consequently, the mortality trends observed in our study are likely predominantly driven by melanoma. The significant decline in mortality observed after 2014, which temporally corresponds to the introduction of immune checkpoint inhibitors and targeted therapies, likely reflects therapeutic advances that primarily benefit melanoma patients, as these agents have shown limited efficacy for NMSC, which is primarily managed through surgical excision (34, 54). Therefore, the temporal turning point identified in our joinpoint regression analysis may predominantly represent changes in melanoma mortality rather than an improvement across all skin cancer subtypes. However, our aggregate mortality data do not allow us to definitively attribute this decline to specific therapeutic interventions or to quantify their relative contributions. For example, among non-White racial/ethnic groups, a greater proportion of skin cancer deaths may be attributable to squamous cell carcinoma (the more lethal NMSC subtype) rather than melanoma, compared to White populations where melanoma dominates mortality statistics (43). The higher mortality observed in elderly populations (65+ years) may also disproportionately reflect NMSC-related deaths, as squamous cell carcinoma incidence increases markedly with cumulative chronic UV exposure and advancing age (9). Additionally, the gender disparity in mortality (male-to-female ratio of 1.99:1) may be influenced by differential subtype compositions between sexes, as males have higher rates of both melanoma and NMSC but the relative contribution of each subtype to the overall gender gap remains unclear in our aggregated analysis (10, 36). Furthermore, the urban-rural and geographic disparities may also have subtype-specific components: rural areas with high proportions of outdoor workers may have a greater burden of NMSC from chronic occupational UV exposure, while geographic variations in indoor tanning practices may differentially affect melanoma rates (48, 49). Future studies utilizing cancer registry data that provide subtype-specific mortality information, such as the Surveillance, Epidemiology, and End Results (SEER) database, would be valuable for disentangling the distinct contributions of melanoma and NMSC to the overall trends and disparities reported here.

This study has several limitations. First, the CDC WONDER database is based on death certificates, which may involve misclassification of causes of death. Specifically, death certificate data is subject to several well-documented sources of coding errors that may affect the accuracy of our mortality estimates. The identification of the underlying cause of death on death certificates relies on the judgment of certifiers (physicians, medical examiners, or coroners), and inaccuracies may arise during this process, particularly among patients with multiple comorbidities where skin malignant neoplasms may not be recognized or recorded as the primary cause of death (6). Furthermore, the translation of cause-of-death information into ICD-10 codes (C43-C44) introduces additional potential for misclassification. Non-melanoma skin cancers (C44), which are less frequently fatal, may be particularly susceptible to underreporting on death certificates, while melanoma (C43) may occasionally be miscoded as metastatic cancer of unknown primary site (55). These coding errors could lead to both underestimation and overestimation of mortality rates. Underestimation may occur when skin malignant neoplasms contribute to but are not listed as the underlying cause of death, whereas overestimation may result from incorrect attribution of deaths to skin malignant neoplasms. Prior studies suggest that the net effect is more likely to be underestimation, particularly for non-melanoma skin cancers, implying that the mortality rates reported in our study may be conservative (8). Importantly, the accuracy of death certificate coding may vary across demographic subgroups including racial/ethnic groups, geographic regions, and urban-rural settings due to differences in access to medical care, availability of dermatopathological expertise, and autopsy rates, which could introduce differential misclassification bias and affect the magnitude of the disparities observed in our study (7). Additionally, over the 22-year study period (1999–2020), changes in coding guidelines, certifier training, and evolving awareness of skin cancer may have influenced coding practices over time, potentially contributing to apparent temporal trends that partially reflect changes in death certificate completion rather than true epidemiological shifts (56).

Second, we were unable to distinguish mortality rate differences between different subtypes of skin malignant neoplasms (such as melanoma versus non-melanoma), which may obscure unique epidemiological characteristics of specific subtypes. This is a particularly important limitation because melanoma and NMSC differ substantially in their etiology, prognosis, treatment response, and demographic distribution. Since melanoma accounts for the vast majority of skin cancer deaths, the aggregate trends and disparities reported in our study predominantly reflect melanoma mortality patterns and may not accurately represent NMSC-specific mortality trends. As discussed above, the observed post-2014 mortality decline is likely driven primarily by melanoma-specific therapeutic advances, and the observed demographic disparities may have different magnitudes or even different directions when examined for each subtype separately. Notably, NMSC deaths particularly those from squamous cell carcinoma may be underrepresented in our analysis due to lower case-fatality rates and potential underreporting on death certificates, further compounding this limitation (6, 55). Nevertheless, combining C43 and C44 codes is a widely adopted methodological approach in population-based mortality studies using death certificate databases, as the CDC WONDER standard query system does not permit separate tabulation of individual ICD-10 subcodes within the broader category of skin malignant neoplasms. Despite this constraint, the overall patterns identified in our study remain informative for understanding the general landscape of skin cancer mortality in the United States, and our findings can serve as a foundation for future subtype-specific investigations using cancer registry databases such as SEER.

Third, this study uses an ecological design and cannot establish causal relationships between observed temporal trends and potential contributing factors such as therapeutic interventions, policy changes, or other concurrent developments. The study lacks adjustment for potential confounding factors such as socioeconomic status, educational level, and health insurance status at the individual level. Consequently, while we observe temporal correlations between mortality trends and various developments (such as the introduction of new therapies), we cannot determine whether these associations reflect direct causal relationships or are mediated by other unmeasured factors. Importantly, this ecological limitation means that the demographic and geographic disparities observed in our study including racial/ethnic differences, urban-rural gaps, and regional variations may be substantially confounded by unmeasured socioeconomic factors that are not captured in death certificate data. Key socioeconomic determinants that likely influence skin cancer outcomes but cannot be controlled for in our analysis include individual and household income, educational attainment, employment status, health insurance coverage, access to dermatological care, and healthcare utilization patterns. For example, the higher mortality rates observed in rural areas and certain Appalachian states may be partially attributed to lower income levels, reduced access to specialized dermatological services, delayed diagnosis due to transportation barriers, and lower rates of health insurance coverage in these populations. Similarly, the racial/ethnic disparities in mortality may be influenced by differential access to preventive care, health insurance status, and socioeconomic resources that affect both risk factors (such as occupational UV exposure) and healthcare-seeking behaviors. Future studies should utilize individual-level data sources, such as cancer registry data linked with census information or population-based cohort studies with detailed socioeconomic measurements, to enable proper adjustment for these important covariates and to better disentangle the independent effects of demographic characteristics from socioeconomic determinants of skin cancer mortality disparities.

Fourth, our study period ends in 2020, which coincides with the onset of the COVID-19 pandemic but does not capture its full impact. The pandemic led to widespread disruptions in dermatological care, including delayed screenings, deferred biopsies, and postponed treatments, which may have resulted in diagnostic delays and stage migration of skin cancers in subsequent years. Moreover, the 2020 mortality data itself may be affected by changes in healthcare utilization and death certification practices during the pandemic. Future studies should extend the analysis beyond 2020 to comprehensively evaluate the impact of the pandemic on skin cancer mortality trends and to determine whether the declining trends observed in our study have been sustained or disrupted. Fifth, our analysis was restricted to adults aged 25 years and older, which limits the generalizability of our findings to younger populations. While this age restriction was methodologically justified due to the extremely low mortality rates from skin malignant neoplasms in individuals under 25 years, it means that our study cannot capture potential trends in pediatric and young adult populations or assess the long-term mortality impact of prevention programs targeting children and adolescents. Given emerging concerns about changing skin cancer patterns in younger age groups, future studies should specifically examine trends in these populations using data sources with sufficient case numbers or alternative analytical approaches designed for rare events. Additionally, our analysis was unable to assess the direct impact of skin cancer screening programs and specific treatment advances on mortality rates.

In conclusion, despite the overall declining trend in skin malignant neoplasm mortality rates in the United States, significant disparities exist among different populations, indicating the need for targeted prevention and intervention strategies to further reduce the burden of skin cancer mortality and decrease health inequalities.

## Data Availability

The original contributions presented in the study are included in the article/**Supplementary Material**. Further inquiries can be directed to the corresponding author.
