# Supplementary material for: Trends and disparities in skin malignant neoplasm mortality in the United States: a 22-year trend analysis based on CDC WONDER Data (1999-2020)
Source: Front Oncol. 2026 Mar 11;16:1770375. doi: 10.3389/fonc.2026.1770375 (PMC13012982; doi:10.3389/fonc.2026.1770375)
Supplement: Supplementary file 1 [file Table1.doc]

**Supplementary Table 1.Age-Adjusted Mortality Rates of Skin Malignant Neoplasms by Gender in the United States, 1999-2020**

| **Year** | **Overall** | **Male** | **Female** |
| --- | --- | --- | --- |
| **1999** | 5.346(5.238-5.453) | 8.094(7.888-8.301) | 3.363(3.249-3.477) |
| **2000** | 5.384(5.276-5.491) | 8.056(7.850-8.261) | 3.435(3.321-3.550) |
| **2001** | 5.461(2.354-5.568) | 8.221(8.016-8.426) | 3.388(3.276-3.500) |
| **2002** | 5.303(5.198-5.408) | 7.996(7.796-8.196) | 3.313(3.202-3.423) |
| **2003** | 5.373(5.268-5.477) | 8.131(7.931-8.331) | 3.369(3.258-3.480) |
| **2004** | 5.333(5.230-5.437) | 8.207(8.008-8.407) | 3.235(3.127-3.342) |
| **2005** | 5.498(5.394-5.602) | 8.349(8.150-8.549) | 3.412(3.302-3.521) |
| **2006** | 5.519(5.416-5.623) | 8.587(8.387-8.787) | 3.274(3.168-3.381) |
| **2007** | 5.5(5.398-5.603) | 8.46(8.264-8.656) | 3.32(3.213-3.427) |
| **2008** | 5.448(5.347-5.549) | 8.464(8.270-8.658) | 3.194(3.090-3.298) |
| **2009** | 5.707(5.604-5.809) | 8.811(8.614-9.007) | 3.428(3.321-3.535) |
| **2010** | 5.591(5.490-5.692) | 8.679(8.486-8.872) | 3.289(3.185-3.393) |
| **2011** | 5.54(5.441-5.640) | 8.628(8.438-8.817) | 3.224(3.122-3.327) |
| **2012** | 5.532(5.434-5.630) | 8.533(8.346-8.720) | 3.285(3.182-3.387) |
| **2013** | 5.548(5.450-5.646) | 8.733(8.546-8.919) | 3.146(3.046-3.245) |
| **2014** | 5.512(5.416-5.608) | 8.624(8.441-8.807) | 3.154(3.056-3.252) |
| **2015** | 5.307(5.214-5.401) | 8.195(8.018-8.371) | 3.102(3.006-3.199) |
| **2016** | 4.888(4.799-5.977) | 7.679(7.510-7.848) | 2.761(2.670-2.851) |
| **2017** | 4.764(4.678-4.850) | 7.408(7.243-7.572) | 2.766(2.676-2.855) |
| **2018** | 4.825(4.739-4.912) | 7.343(7.182-7.504) | 2.894(2.803-2.985) |
| **2019** | 4.634(4.550-4.718) | 7.066(6.910-7.223) | 2.754(2.666-2.842) |
| **2020** | 4.667(4.584-4.751) | 7.079(6.924-7.234) | 2.777(2.690-2.865) |
| **Total** | 5.299(5.278-5.320) | 8.137(8.097-8177) | 3.157(3.135-3.179) |

**Age-Adjusted Rate (95% CI).This table presents the age-adjusted mortality rates (per 100,000 population) and 95% confidence intervals for skin malignant neoplasms during 1999-2020, stratified by year and gender based on CDC WONDER database. The table includes three columns: overall population, male, and female, displaying annual mortality rates over the 22-year period and gender-specific disparities. The final row presents the overall age-adjusted mortality rate for the entire study period. The table enables direct comparison of mortality gaps between males and females and their temporal variations.**

**Supplementary Table 2.Annual Age-Adjusted Mortality Rates of Skin Malignant Neoplasms by Age Group in the United States, 1999-2020**

| **Year** | **25-44** | **45-64** | **65+** |
| --- | --- | --- | --- |
| **1999** | 1.04(0.972-1.109) | 4.843(4.667-5.019) | 16.419(15.992-16.846) |
| **2000** | 1.014(0.946-1.081) | 4.85(4.677-5.024) | 16.506(16.080-16.932) |
| **2001** | 1.022(0.954-1.090) | 4.863(4.692-5.033) | 16.871(16.442-17.300) |
| **2002** | 1.023(0.954-1.091) | 4.557(4.395-4.719) | 16.728(16.303-17.153) |
| **2003** | 0.984(0.917-1.052) | 4.774(4.611-4.937) | 16.829(16.406-17.253) |
| **2004** | 0.922(0.857-0.987) | 4.638(4.480-4.797) | 17.002(16.578-17.425) |
| **2005** | 0.959(0.893-1.026) | 4.557(4.402-4.712) | 17.944(17.512-18.376) |
| **2006** | 0.889(0.825-0.954) | 4.599(4.445-4.752) | 18.167(17.736-18.599) |
| **2007** | 0.831(0.769-0.894) | 4.661(4.508-4.813) | 18.129(17.702-18.557) |
| **2008** | 0.784(0.724-0.844) | 4.482(4.334-4.630) | 18.306(17.880-18.832) |
| **2009** | 0.912(0.847-0.978) | 4.627(4.478-4.776) | 19.196(18.764-19.628) |
| **2010** | 0.799(0.738-0.860) | 4.579(4.432-4.725) | 18.983(18.557-19.410) |
| **2011** | 0.762(0.702-0.822) | 4.361(4.219-4.503) | 19.198(18.774-19.622) |
| **2012** | 0.749(0.690-0.807) | 4.358(4.216-4.500) | 19.193(18.775-19.611) |
| **2013** | 0.81(0.749-0.871) | 4.251(4.111-4.391) | 19.354(18.939-19.768) |
| **2014** | 0.719(0.661-0.776) | 4.289(4.149-4.430) | 19.466(19.057-19.875) |
| **2015** | 0.66(0.605-0.714) | 3.992(3.857-4.127) | 19.014(18.615-19.413) |
| **2016** | 0.587(0.536-0.639) | 3.666(3.537-3.796) | 17.557(17.178-17.935) |
| **2017** | 0.564(0.514-0.614) | 3.601(3.473-3.729) | 17.263(16.892-17.633) |
| **2018** | 0.56(0.510-0.610) | 3.515(3.388-3.642) | 17.587(17.218-17.955) |
| **2019** | 0.508(0.461-0.555) | 3.436(3.310-3.562) | 16.948(16.592-17.304) |
| **2020** | 0.543(0.494-0.592) | 3.418(3.292-3.544) | 17.061(16.708-17.413) |
| **Total** | 0.8(0.787-0.813) | 4.279(4.248-4.310) | 17.902(17.816-17.989) |

**Age-Adjusted Rate (95% CI).This table presents the annual age-adjusted mortality rates (per 100,000 population) with 95% confidence intervals for skin malignant neoplasms during 1999-2020, stratified by year and age group based on CDC WONDER database. The table includes three age groups: 25-44 years, 45-64 years, and 65 years and older, displaying year-specific mortality rates and age-related disparities over the 22-year period. The final row labeled "Total" presents the overall age-adjusted mortality rate with 95% confidence intervals for each age group across the entire study period. The table enables direct comparison of mortality gaps across age groups (elderly population showing significantly higher mortality than younger and middle-aged groups) and their temporal variations.**

**Supplementary Table 3.Annual Age-Adjusted Mortality Rates of Skin Malignant Neoplasms by Race/Ethnicity in the United States, 1999-2020**

| **Year** | **Hispanic or Latino** | **Black or African American** | **White people** |
| --- | --- | --- | --- |
| **1999** | 1.002(0.738-1.328) | 1.459(1.272-1.647) | 5.936(5.814-6.057) |
| **2000** | 1.113(0.849-1.432) | 1.733(1.529-1.937) | 5.93(5.809-6.051) |
| **2001** | 0.988(0.746-1.283) | 1.509(1.320-1.698) | 6.061(5.940-6.182) |
| **2002** | 1.064(0.823-1.354) | 1.455(1.274-1.636) | 5.878(5.760-5.996) |
| **2003** | 1.1(0.854-1.395) | 1.401(1.222-1.579) | 6.015(5.896-6.134) |
| **2004** | 0.779(0.580-1.024) | 1.393(1.216-1.569) | 5.996(5.877-6.114) |
| **2005** | 1.256(1.001-1.511) | 1.439(1.261-1.617) | 6.172(6.053-6.291) |
| **2006** | 1.272(1.012-1.532) | 1.367(1.195-1.538) | 6.204(6.086-6.322) |
| **2007** | 1.148(0.914-1.382) | 1.54(1.362-1.718) | 6.155(6.038-6.272) |
| **2008** | 0.756(0.585-0.962) | 1.335(1.174-1.497) | 6.139(6.023-6.255) |
| **2009** | 1.123(0.904-1.343) | 1.304(1.144-1.463) | 6.499(6.380-6.618) |
| **2010** | 1.128(0.912-1.344) | 1.214(1.065-1.364) | 6.34(6.224-6.456) |
| **2011** | 1.033(0.833-1.232) | 1.284(1.130-1.438) | 6.305(6.190-6.420) |
| **2012** | 1.053(0.858-1.248) | 1.157(1.014-1.301) | 6.299(6.185-6.413) |
| **2013** | 0.926(0.748-1.104) | 1.233(1.089-1.376) | 6.356(6.242-6.470) |
| **2014** | 1.055(0.871-1.238) | 1.339(1.189-1.489) | 6.32(6.208-6.433) |
| **2015** | 0.934(0.768-1.100) | 1.178(1.040-1.316) | 6.114(6.004-6.224) |
| **2016** | 1.009(0.839-1.180) | 0.988(0.863-1.113) | 5.627(5.522-5.731) |
| **2017** | 0.894(0.740-1.048) | 1.092(0.964-1.220) | 5.507(5.405-5.610) |
| **2018** | 0.909(0.756-1.062) | 1.131(1.001-1.261) | 5.561(5.459-5.664) |
| **2019** | 0.843(0.702-0.983) | 1.08(0.956-1.204) | 5.333(5.234-5.432) |
| **2020** | 1.035(0.880-1.190) | 1.08(0.957-1.202) | 5.398(5.299-5.497) |
| **Total** | 1.018(0.975-1.060) | 1.291(1.258-1.324) | 5.996(5.972-6.020) |

**Age-Adjusted Rate (95% CI).This table presents the annual age-adjusted mortality rates (per 100,000 population) with 95% confidence intervals for skin malignant neoplasms during 1999-2020, stratified by year and race/ethnicity based on CDC WONDER database. The table includes three racial/ethnic categories: Hispanic or Latino, Black or African American, and White people, displaying year-specific mortality rates and racial/ethnic disparities over the 22-year period. The final row labeled "Total" presents the overall age-adjusted mortality rate with 95% confidence intervals for the entire study period. The table enables direct comparison of mortality disparities across racial/ethnic groups (White population showing significantly higher mortality than Black and Hispanic populations) and their temporal variations across the two decades.**

**Supplementary Table 4.Annual Age-Adjusted Mortality Rates of Skin Malignant Neoplasms by Census Region in the United States, 1999-2020**

| **Census Region** | **Year** | **Age-Adjusted Rate with 95% CI** |
| --- | --- | --- |
| **Census Region 1: Northeast** | 1999 | 4.933(4.705-5.160) |
| **Census Region 1: Northeast** | 2000 | 5.077(4.847-5.307) |
| **Census Region 1: Northeast** | 2001 | 5.349(5.115-5.584) |
| **Census Region 1: Northeast** | 2002 | 5.034(4.809-5.260) |
| **Census Region 1: Northeast** | 2003 | 5.124(4.897-5.351) |
| **Census Region 1: Northeast** | 2004 | 5.112(4.887-5.337) |
| **Census Region 1: Northeast** | 2005 | 5.311(5.082-5.540) |
| **Census Region 1: Northeast** | 2006 | 5.23(5.004-5.457) |
| **Census Region 1: Northeast** | 2007 | 5.064(4.842-5.285) |
| **Census Region 1: Northeast** | 2008 | 5.05(4.830-5.271) |
| **Census Region 1: Northeast** | 2009 | 5.284(5.060-5.508) |
| **Census Region 1: Northeast** | 2010 | 5.175(4.954-5.395) |
| **Census Region 1: Northeast** | 2011 | 5.096(4.880-5.313) |
| **Census Region 1: Northeast** | 2012 | 5.196(4.979-5.414) |
| **Census Region 1: Northeast** | 2013 | 5.068(4.853-5.283) |
| **Census Region 1: Northeast** | 2014 | 5.08(4.867-5.292) |
| **Census Region 1: Northeast** | 2015 | 4.634(4.432-4.836) |
| **Census Region 1: Northeast** | 2016 | 4.488(4.290-4.686) |
| **Census Region 1: Northeast** | 2017 | 4.387(4.194-4.581) |
| **Census Region 1: Northeast** | 2018 | 4.295(4.106-4.484) |
| **Census Region 1: Northeast** | 2019 | 4.117(3.932-4.302) |
| **Census Region 1: Northeast** | 2020 | 4.261(4.073-4.448) |
| **Census Region 1: Northeast** | Total | 4.93(4.884-4.976) |
| **Census Region 2: Midwest** | 1999 | 5.255(5.035-5.475) |
| **Census Region 2: Midwest** | 2000 | 5.158(4.941-5.375) |
| **Census Region 2: Midwest** | 2001 | 4.982(4.771-5.194) |
| **Census Region 2: Midwest** | 2002 | 5.058(4.845-5.270) |
| **Census Region 2: Midwest** | 2003 | 4.927(4.719-5.135) |
| **Census Region 2: Midwest** | 2004 | 5.128(4.917-5.339) |
| **Census Region 2: Midwest** | 2005 | 5.398(5.183-5.614) |
| **Census Region 2: Midwest** | 2006 | 5.369(5.155-5.583) |
| **Census Region 2: Midwest** | 2007 | 5.233(5.024-5.442) |
| **Census Region 2: Midwest** | 2008 | 5.291(5.082-5.500) |
| **Census Region 2: Midwest** | 2009 | 5.656(5.440-5.872) |
| **Census Region 2: Midwest** | 2010 | 5.606(5.392-5.821) |
| **Census Region 2: Midwest** | 2011 | 5.54(5.328-5.752) |
| **Census Region 2: Midwest** | 2012 | 5.469(5.261-5.677) |
| **Census Region 2: Midwest** | 2013 | 5.623(5.412-5.833) |
| **Census Region 2: Midwest** | 2014 | 5.537(5.330-5.744) |
| **Census Region 2: Midwest** | 2015 | 5.443(5.239-5.647) |
| **Census Region 2: Midwest** | 2016 | 4.978(4.785-5.171) |
| **Census Region 2: Midwest** | 2017 | 4.85(4.661-5.039) |
| **Census Region 2: Midwest** | 2018 | 4.943(4.753-5.132) |
| **Census Region 2: Midwest** | 2019 | 4.676(4.493-4.859) |
| **Census Region 2: Midwest** | 2020 | 4.97(4.782-5.159) |
| **Census Region 2: Midwest** | Total | 5.224(5.180-5.268) |
| **Census Region 3: South** | 1999 | 5.623(5.437-5.808) |
| **Census Region 3: South** | 2000 | 5.661(5.476-5.846) |
| **Census Region 3: South** | 2001 | 5.816(5.631-6.002) |
| **Census Region 3: South** | 2002 | 5.593(5.412-5.773) |
| **Census Region 3: South** | 2003 | 5.635(5.455-5.814) |
| **Census Region 3: South** | 2004 | 5.511(5.335-5.687) |
| **Census Region 3: South** | 2005 | 5.702(5.524-5.879) |
| **Census Region 3: South** | 2006 | 5.721(5.546-5.897) |
| **Census Region 3: South** | 2007 | 5.804(5.629-5.979) |
| **Census Region 3: South** | 2008 | 5.654(5.483-5.825) |
| **Census Region 3: South** | 2009 | 5.823(5.651-5.995) |
| **Census Region 3: South** | 2010 | 5.698(5.529-5.866) |
| **Census Region 3: South** | 2011 | 5.704(5.537-5.870) |
| **Census Region 3: South** | 2012 | 5.627(5.463-5.791) |
| **Census Region 3: South** | 2013 | 5.774(5.610-5.938) |
| **Census Region 3: South** | 2014 | 5.734(5.572-5.895) |
| **Census Region 3: South** | 2015 | 5.457(5.302-5.612) |
| **Census Region 3: South** | 2016 | 4.978(4.831-5.124) |
| **Census Region 3: South** | 2017 | 4.876(4.732-5.019) |
| **Census Region 3: South** | 2018 | 4.964(4.820-5.108) |
| **Census Region 3: South** | 2019 | 4.769(4.631-4.907) |
| **Census Region 3: South** | 2020 | 4.652(4.516-4.787) |
| **Census Region 3: South** | Total | 5.452(5.418-5.487) |
| **Census Region 4: West** | 1999 | 5.374(5.134-5.613) |
| **Census Region 4: West** | 2000 | 5.416(5.177-5.654) |
| **Census Region 4: West** | 2001 | 5.413(5.178-5.649) |
| **Census Region 4: West** | 2002 | 5.362(5.130-5.593) |
| **Census Region 4: West** | 2003 | 5.641(5.406-5.875) |
| **Census Region 4: West** | 2004 | 5.42(5.192-5.648) |
| **Census Region 4: West** | 2005 | 5.423(5.198-5.649) |
| **Census Region 4: West** | 2006 | 5.584(5.357-5.810) |
| **Census Region 4: West** | 2007 | 5.586(5.362-5.809) |
| **Census Region 4: West** | 2008 | 5.591(5.370-5.812) |
| **Census Region 4: West** | 2009 | 6.002(5.775-6.229) |
| **Census Region 4: West** | 2010 | 5.84(5.618-6.062) |
| **Census Region 4: West** | 2011 | 5.649(5.433-5.864) |
| **Census Region 4: West** | 2012 | 5.705(5.491-5.919) |
| **Census Region 4: West** | 2013 | 5.519(5.312-5.726) |
| **Census Region 4: West** | 2014 | 5.574(5.369-5.779) |
| **Census Region 4: West** | 2015 | 5.53(5.328-5.732) |
| **Census Region 4: West** | 2016 | 4.943(4.755-5.132) |
| **Census Region 4: West** | 2017 | 4.865(4.680-5.049) |
| **Census Region 4: West** | 2018 | 4.877(4.694-5.059) |
| **Census Region 4: West** | 2019 | 4.762(4.583-4.941) |
| **Census Region 4: West** | 2020 | 4.702(4.527-4.877) |
| **Census Region 4: West** | Total | 5.393(5.348-5.438) |

**Age-Adjusted Rate (95% CI).This table presents the annual age-adjusted mortality rates (per 100,000 population) with 95% confidence intervals for skin malignant neoplasms during 1999-2020, stratified by year and census region based on CDC WONDER database. The table includes four census regions: Census Region 1 (Northeast), Census Region 2 (Midwest), Census Region 3 (South), and Census Region 4 (West), displaying year-specific mortality rates and regional disparities over the 22-year period. The final row labeled "Total" for each region presents the overall age-adjusted mortality rate with 95% confidence intervals for the entire study period. The table enables direct comparison of mortality disparities across geographic regions and their temporal variations across the two decades.**

**Supplementary Table 5.Annual Age-Adjusted Mortality Rates of Skin Malignant Neoplasms by Urbanization Level in the United States, 1999-2020**

| **Year** | **Metropolitan** | **Non-Metropolitan** |
| --- | --- | --- |
| **1999** | 5.285(5.166-5.403) | 5.701(5.439-5.963) |
| **2000** | 5.298(5.180-5.415) | 5.656(5.396-5.915) |
| **2001** | 5.321(5.204-5.438) | 6.038(5.771-6.305) |
| **2002** | 5.208(5.094-5.323) | 5.7(5.443-5.958) |
| **2003** | 5.266(5.152-5.380) | 5.898(5.637-6.159) |
| **2004** | 5.185(5.072-5.297) | 5.978(5.716-6.239) |
| **2005** | 5.359(5.246-5.473) | 6.06(5.798-6.322) |
| **2006** | 5.445(5.331-5.558) | 5.892(5.636-6.147) |
| **2007** | 5.386(5.275-5.498) | 6.021(5.765-6.278) |
| **2008** | 5.314(5.205-5.424) | 6.003(5.747-6.258) |
| **2009** | 5.608(5.495-5.720) | 6.372(6.110-6.634) |
| **2010** | 5.494(5.384-5.604) | 6.13(5.874-6.386) |
| **2011** | 5.432(5.324-5.541) | 6.12(5.865-6.374) |
| **2012** | 5.431(5.324-5.538) | 6.064(5.811-6.316) |
| **2013** | 5.383(5.277-5.488) | 6.366(6.107-6.624) |
| **2014** | 5.409(5.304-5.514) | 6.314(6.059-6.569) |
| **2015** | 5.136(5.035-5.237) | 6.342(6.087-6.596) |
| **2016** | 4.714(4.619-4.810) | 5.818(5.574-6.062) |
| **2017** | 4.611(4.518-4.704) | 5.691(5.454-5.929) |
| **2018** | 4.655(4.562-4.747) | 5.631(5.395-5.867) |
| **2019** | 4.467(4.378-4.557) | 5.442(5.212-5.672) |
| **2020** | 4.457(4.368-4.546) | 5.745(5.510-5.980) |
| **Total** | 5.16(5.137-5.182) | 5.96(5.907-6.014) |

**Age-Adjusted Rate (95% CI).This table presents the annual age-adjusted mortality rates (per 100,000 population) with 95% confidence intervals for skin malignant neoplasms during 1999-2020, stratified by year and urbanization level based on CDC WONDER database. The table includes two geographic categories: Metropolitan and Non-Metropolitan areas, displaying year-specific mortality rates and urban-rural disparities over the 22-year period. The final row labeled "Total" presents the overall age-adjusted mortality rate with 95% confidence intervals for the entire study period by urbanization level. The table enables direct comparison of mortality disparities between urban and rural areas (Non-Metropolitan areas showing consistently higher mortality than Metropolitan areas) and their temporal variations across the two decades.**

**Supplementary Table 6.Age-Adjusted Mortality Rates of Skin Malignant Neoplasms by State in the United States, 1999-2020**

| **State** | **Age-Adjusted Rate with 95% CI** |
| --- | --- |
| **Idaho** | 6.792(6.449-7.134) |
| **Oklahoma** | 6.742(6.528-6.955) |
| **West Virginia** | 6.511(6.229-6.794) |
| **Kentucky** | 6.467(6.272-6.661) |
| **Delaware** | 6.421(6.007-6.836) |
| **Tennessee** | 6.403(6.243-6.563) |
| **Utah** | 6.23(5.948-6.513) |
| **Colorado** | 6.033(5.847-6.219) |
| **Kansas** | 6.005(5.777-6.234) |
| **Wyoming** | 5.948(5.421-6.476) |
| **New Hampshire** | 5.936(5.606-6.265) |
| **Oregon** | 5.906(5.714-6.099) |
| **Missouri** | 5.897(5.742-6.052) |
| **Arizona** | 5.894(5.742-6.046) |
| **Maine** | 5.87(5.561-6.178) |
| **Indiana** | 5.85(5.698-6.001) |
| **Nevada** | 5.829(5.581-6.078) |
| **Vermont** | 5.817(5.354-6.281) |
| **Nebraska** | 5.793(5.514-6.072) |
| **Washington** | 5.784(5.634-5.934) |
| **North Carolina** | 5.678(5.553-5.802) |
| **Florida** | 5.677(5.598-5.756) |
| **Iowa** | 5.543(5.338-5.748) |
| **Montana** | 5.528(5.168-5.887) |
| **Massachusetts** | 5.526(5.386-5.667) |
| **Ohio** | 5.489(5.382-5.595) |
| **Rhode Island** | 5.486(5.145-5.826) |
| **Virginia** | 5.46(5.325-5.595) |
| **South Carolina** | 5.439(5.266-5.611) |
| **Alabama** | 5.43(5.262-5.599) |
| **Pennsylvania** | 5.396(5.299-5.493) |
| **Total** | 5.299(5.278-5.320) |
| **South Dakota** | 5.297(4.903-5.691) |
| **Arkansas** | 5.279(5.069-5.490) |
| **New Mexico** | 5.223(4.966-5.480) |
| **Wisconsin** | 5.112(4.966-5.259) |
| **Texas** | 5.09(5.011-5.168) |
| **California** | 5.024(4.963-5.085) |
| **Maryland** | 4.939(4.789-5.089) |
| **Georgia** | 4.898(4.774-5.021) |
| **Mississippi** | 4.83(4.623-5.036) |
| **Connecticut** | 4.812(4.636-4.988) |
| **Michigan** | 4.806(4.698-4.915) |
| **New Jersey** | 4.789(4.675-4.904) |
| **Illinois** | 4.71(4.613-4.808) |
| **Minnesota** | 4.704(4.555-4.853) |
| **Louisiana** | 4.396(4.235-4.557) |
| **New York** | 4.203(4.131-4.276) |
| **Alaska** | 4.15(3.660-4.640) |
| **North Dakota** | 3.988(3.617-4.358) |
| **Hawaii** | 3.389(3.149-3.630) |
| **District of Columbia** | 2.848(2.487-3.208) |

**Age-Adjusted Rate (95% CI).This table presents the age-adjusted mortality rates (per 100,000 population) with 95% confidence intervals for skin malignant neoplasms across all 50 U.S. states and the District of Columbia during 1999-2020, ranked from highest to lowest mortality based on CDC WONDER database. Idaho had the highest mortality rate (6.792, 95% CI: 6.449-7.134), while the District of Columbia had the lowest (2.848, 95% CI: 2.487-3.208), with the overall national rate at 5.299 (95% CI: 5.278-5.320). The table reveals substantial geographic disparities in skin malignant neoplasm mortality, with higher mortality states predominantly located in the Western, South-Central, and Appalachian regions, while lower mortality states are mainly concentrated in densely populated Northeastern areas.**
